# Concerns regarding the pediatric workforce: what are we missing?

**DOI:** 10.1038/s41390-025-04527-7

**Published:** 2025-10-30

**Authors:** Gary L. Freed

**Affiliations:** Susan B. Meister Child Health Evaluation and Research Center, University of Michigan, Ann Arbor, MI USA

## Abstract

**Abstract:**

Concerns about the adequacy of the pediatric physician workforce have intensified in recent years, yet many proposed solutions may not address some of the most critical underlying issues. This manuscript challenges prevailing assumptions—particularly the emphasis on financial compensation as a primary deterrent to entering pediatrics—and recommends a broader, evidence-based exploration of factors influencing specialty and subspecialty choice. While economic disparities between pediatricians and adult care providers are real and must be addressed, other influences may play a significant role in specialty selection among medical students and subspecialties among pediatric residents. There is a need to go beyond the study of those who chose pediatrics and turn instead to those who specifically did not choose pediatrics (and by extension chose other specialties). To grow the specialty, we need to find out what, precisely, would have made pediatrics, or one of the pediatric subspecialties, more attractive to them. We must also begin to grapple with the growing gender imbalance in the specialty and its impact on workforce adequacy.

**Impact:**

- It is more helpful to look at longer term trends than focusing on yearly fluctuations in match rates.
- It may be helpful to learn from those who did not choose pediatrics what influenced that decision.
- There may be modifiable and non-modifiable inherent aspects to pediatrics which make it less attractive to some medical students.
- From a workforce perspective, there is a need to address the gender imbalance in pediatrics.
- There is a need to set workforce goals, not only to find problems.

## Influences on specialty decision-making

Several recent reports have highlighted concerns regarding the pediatric workforce and raised alarms at the recent trends in residency and subspecialty match rates.^1–3^ As part of these discussions, much attention is focused on compensation. Previous studies have demonstrated both the relative and absolute negative financial renumeration of pediatricians compared to physicians who provide care for adults.^4,5^ These differences are the result of unfair structural deficiencies in the health care payment system and can only be addressed through policy and regulatory changes at federal and state levels.^6–8^ Sadly, advocacy in this realm has been ongoing for several decades and has met with limited success. Despite pronouncements to the contrary, our society does not value the provision of health care to children (in monetary terms) at the same level as the care of adults. Efforts in this arena must continue but other influences on the pediatric physician workforce also must be examined and addressed concurrently.

These financial disparities among specialties have often been declared by many pediatric leaders to be a primary causal factor in the decision of trainees to pursue, or not pursue, pediatric residency training and to impact the choice of specific pediatric subspecialties.^9–11^ Undoubtedly, economic considerations affect many aspects of decisions regarding careers. However, the relative importance of economic considerations *at the time of the decision* to become a pediatrician or a specific pediatric subspecialist is something to be more thoroughly explored, not a condition to be assumed.

It is important to note that the published expressions of significant dissatisfaction with pediatric renumeration have been conducted among those already having completed training---and currently experiencing the frustration of being paid less than their peers who provide care to adults.^11,12^ In contrast, studies of those currently in pediatric training tend to rate compensation relatively low in the relative importance of factors affecting their choice of specialty and/or subspecialty.^13–16^ Thus, while compensation is important, it is likely that many other factors affect the choice of specialty and subspecialty across the fields of medicine and should be explored rigorously.

Because there are a fixed number of graduates from allopathic and osteopathic medical schools in the United States each year, efforts to increase the number of trainees selecting one specialty as their “first choice” will decrease the number of trainees choosing another. This is the nature of a zero-sum game. In plain terms, for the field of pediatrics to grow, pediatrics must attract some medical school graduates currently choosing other specialties or attract a higher proportion from new medical schools or those expanding class size. However, to date, recent efforts by pediatric leaders and organizations to understand specialty choice have heretofore been almost exclusively directed at those already in the pediatric pipeline (e.g., those in pediatric training or pediatric practice). Essentially, we ask those that have already chosen pediatrics (and by extension, not having chosen other specialties), why they have chosen pediatrics.^13–17^ This strategy will not tell us what needs to be done to attract more potential pediatricians, but rather only provide information regarding what we have done to attract a perceived insufficient number of trainees.

One of the only studies of current medical students found financial issues were a factor more commonly expressed as influencing specialty choice by those not pursuing pediatrics, but its importance relative to other factors was not assessed. This study also assessed medical school experiences students reported as influential in choosing their specialty and found little difference between those choosing pediatrics or other specialties.^17^ Another potentially helpful strategy would be to further study medical students who specifically did *not* choose pediatrics to determine what if anything, precisely, would have made pediatrics more attractive to them, or if they perceived specific characteristics of the profession to not be desirable. I hypothesize such an effort would identify some modifiable and some non-modifiable factors. For example, if we find that a significant factor for many trainees is the desire to perform procedures on a routine basis, that will be inconsistent with both general pediatrics and most pediatric subspecialties. No amount of pizza parties, pediatric interest groups or loan repayments will change that. Although there are a few pediatric subspecialties that would fit that desire, they are the subspecialties that currently already fill their match lists. Similarly, if we find many who chose a different specialty because of the attractiveness of shift work with no continuity of care, it will also rule out much of pediatrics as we know it today but may speak to which aspects of pediatrics might be at least partially modifiable to medical school graduates in future years.

However, we may find that some have not chosen pediatrics because of unsatisfying experiences in classroom didactics or clinical experiences with the care of children.^3^ There may indeed be curricular or experiential issues to which we can devote energy to address, especially when armed with data.

Another strategy to consider is to assess, over time, the proportion of medical school graduates at each US medical school who pursue pediatrics residency training. For those schools (both allopathic and osteopathic) that produce either a greater proportion or greater number of future pediatricians than heir peers, a deeper dive into their actions, culture and environment may provide actionable ideas for other pediatric departments.

## Lack of gender balance in the pediatric workforce

One important issue that is infrequently addressed in discussions of the pediatric workforce is the relative paucity of men entering the field. Although the absolute number of women entering pediatric training over the past decade has increased by >10%, there has been a decrease in the number of men. In fact, there actually were fewer men in absolute numbers entering pediatric training in 2024 compared with 2010.^18^ If similar numbers of men sought to enter pediatric training as the current number of women, there would be a marked surplus of applicants for currently available residency positions and subsequently for all available subspecialty openings. Pediatrics would be similar to specialties like dermatology and ophthalmology for which there is competition for scarce positions.

Pointing out this reality should not be interpreted as a mechanism or desire to decrease the number of women in pediatrics or to suggest misogynistic intent. Nor does this reality negate the importance of gender equity in pay and leadership roles in pediatrics.^19–21^ Rather, by facing the reality of the current gender imbalance in the pediatrics workforce, we can consider additional strategies that may have a great likelihood of increasing the profession’s footprint.

Although many have hypothesized why such a gender imbalance has developed in pediatrics, focused studies of male medical students who have chosen fields other than pediatrics have not been conducted. Like the strategy suggested above, such investigations may uncover both modifiable and non-modifiable issues. Financial renumeration may, or may not, be a dominant factor. The only current certainty is that such information is sorely missing from the efforts to develop effective strategies to increase the pediatric workforce.

## The importance of taking the long view in workforce planning

There are year-to-year fluctuations in match rates for specific specialties and subspecialties. Because there are many factors that can impact the workforce on an annual basis, it is rarely helpful to react strongly to a single year of data. Rather, trends over time provide for a more rational basis for policy change or major advocacy efforts. For example, in 2024 compared to 2023, there was a decrease of 73 residency positions filled in the pediatric match. This decrease was the focus of significant concern within the pediatric community, including predictions that this foretold a crisis up to and including the future demise of the profession.^2,4^

However, the following year (2025) pediatric residency match resulted in >95% of positions filled (compared to 91% in 2024) despite an overall *increase* in the number of positions offered. In fact, 2025 saw the largest number of medical school graduates ever matching in pediatrics; 3043 in 2025 vs. 2887 in 2024.^22^ When looking at long-term trends in the field, over the last 15 years there has been an increase of 20% in the absolute number of residents entering pediatrics.^18^

Because of the potential for such annual fluctuation, the temptation to inflate the importance of yearly variation should be tempered, regardless of whether it is consistent with or contrary to the current zeitgeist in the field. Rather, appreciation for multi-year trends, and rigorous assessment of their underlying causes, will likely best serve the long-term interests of the profession.

Additionally, although great interest and attention is placed on match rates for both residency and fellowship training, it is important to keep in mind that they are inaccurate measures of actual matriculants and subject to the variation in the number of positions offered each year. This variation can create a paradoxical situation whereby increasing numbers of available positions may result in lower match rates despite increases in the actual number of individuals beginning training in a given year. Much better measures are either the “fill rate” or the actual numbers of matriculants available through the American Board of Pediatrics.^23^

## The path forward

In the coming year, as many organizations prioritize efforts to address the pediatric workforce, it will be essential that specific goals, in both the academic and private sectors, be clearly articulated. As increasing numbers of pediatric subspecialists enter private practice, focusing only on academic components of the workforce may miss larger trends in play.^24^ Whether the stated workforce goals are framed as the absolute number of training positions available, the number of new matriculants into pediatric residency or subspecialty training, the number of effective clinical full-time equivalents, or something else---they should be informed by data, and clearly expressed. Otherwise, both the leaders of the profession as well as policymakers will not be able to plan and conduct effective strategies to address the legitimate workforce concerns that are raised. Simply put, if we do not know where we want to go, any road will take us there. It is time for our leaders to move past the sounding of the alarm to the time of determining where the profession needs to be, and putting plans in place to get us there--- so that we will all know when we arrive.
